# High-Intensity Focused Ultrasound Ablation for Primary or Salvage Prostate Cancer Therapy: Initial Outcomes in the Veteran Healthcare Setting

**DOI:** 10.3390/life15010017

**Published:** 2024-12-27

**Authors:** Sagar Patel, Ali Antar, Aly Alrabaa, Gal Saffati, Broderick Fleming, Neel Srikishen, Jeremy Slawin, Jennifer Taylor, Jeffrey Jones

**Affiliations:** 1 Operative Care Line, Urology Section, Michael E. DeBakey Veteran Affairs Medical Center, Houston, TX 77030, USA; 2 Scott Department of Urology, Baylor College of Medicine, Houston, TX 77030, USA; 3 Department of Urology, University of Wisconsin School of Medicine and Public Health, Madison, WI 53705, USA; 4 College of Natural Sciences and Mathematics, University of Houston, Houston, TX 77004, USA

**Keywords:** high-intensity focused ultrasound, prostate cancer, veterans’ health, primary therapy, salvage therapy, focal therapy, functional outcomes

## Abstract

High-Intensity Focused Ultrasound (HIFU) provides comparable oncologic, erectile, and urinary outcomes to standard-of-care options for localized prostate cancer. This study reports the largest United States series of HIFU in veterans for both primary and salvage therapies. We retrospectively analyzed the outcomes of 43 veterans treated at the Michael E. DeBakey Veterans Affairs Medical Center from 2018 to 2022. Primary endpoints included prostate-specific antigen (PSA) reduction and local recurrence rates. Secondary endpoints included 30-day complications, Sexual Health Inventory for Men (SHIM), and American Urological Association Symptom Score (AUASS). In our study, 31 veterans (72.1%) received primary treatment and 12 (27.9%) received salvage therapy, with a median follow-up of 23 and 25 months, respectively. Median PSA nadir was 0.16 for primary and 0.12 for salvage groups, with PSA reduction stable over 30 months. Local recurrence occurred in 16.1% of primary and 16.6% of salvage patients. SHIM scores and AUASS were not statistically different before and after HIFU therapy. Short- and intermediate-term results suggest HIFU is a safe and effective treatment option with excellent potency and preserved urinary function, as well as adequate oncological control for primary and salvage therapies for localized prostate cancer in veterans.

## 1. Introduction

Prostate cancer (PCa) is the most common non-skin cancer in men and ranks as the second leading cause of cancer-related deaths among men [1]. In 2023, an estimated 288,300 new cases of PCa were diagnosed, and 34,700 mortalities were reported in the United States alone [1]. The National Cancer Institute projects that 299,010 new cases and 35,250 deaths will occur in 2024 [2]. Thus, PCa remains a significant public health concern, emphasizing the need for early detection and the development of more effective treatment strategies.

Current treatment options for PCa are extensive and continue to advance, including proton beam and intensity-modulated radiation therapy (IMRT), robotic-assisted laparoscopic prostatectomy (RALP), irreversible electroporation, photodynamic therapy, primary cryotherapy, focal laser ablation, and active surveillance [3,4,5,6,7,8]. Among these, High-Intensity Focused Ultrasound (HIFU) ablation, where malignant tissue is significantly heated using targeted ultrasound to promote coagulative necrosis, has emerged as a promising treatment option for localized PCa [9]. One of the reasons for this is the preservation of quality of life following HIFU treatment compared to other treatment options. Although radical prostatectomy or external radiotherapy tends to provide better long-term oncological outcomes, data suggest that HIFU achieves better functional outcomes [10,11]. However, due to limited long-term data and the lack of comparative outcome analysis, the American Urological Association (AUA) and the National Comprehensive Cancer Network (NCCN) currently do not support HIFU as a standard-of-care treatment. Rather, the guidelines recommend HIFU within the context of a research protocol to further evaluate its efficacy and safety in the focal treatment of localized PCa [12].

In 2015, the Food and Drug Administration approved HIFU for prostatic tissue ablation in the United States. Since then, studies have demonstrated that HIFU achieves oncological and functional outcomes comparable to standard-of-care treatments for localized prostate cancer, such as radical prostatectomy and radiation therapy [13,14,15]. Similarly, the use of HIFU in primary whole-gland PCa management has proven to yield satisfactory cancer control with minimal side effects [16]. A prospective trial examining the 3-year outcomes of focal HIFU treatment for low-to-intermediate-risk PCa reported favorable functional outcomes, preservation of quality of life, minimal adverse events, and a low incidence of treatment failure [17]. These findings are further supported by a phase 2 clinical trial demonstrating HIFU’s efficacy as a treatment option for men with intermediate-risk PCa [18].

Additionally, studies show that HIFU is effective in the salvage setting for the management of radiation failure [13,19,20]. A meta-regression analysis found no significant difference in oncological outcomes between salvage radical prostatectomy (SRP) and Salvage High-Intensity Focused Ultrasound (SHIFU) cohorts. However, patients treated with SRP experienced a higher incidence of incontinence compared to those treated with SHIFU [13]. Given its strong oncological control rates, excellent functional outcomes, and preservation of quality of life, HIFU is becoming increasingly recognized as a compelling option for the focal treatment of PCa in both primary and salvage settings [21].

HIFU’s minimally invasive nature and ability to deliver precise, highly localized ablative therapy offers unique advantages, particularly for patients with higher comorbidity indices, a history of abdominal surgery, or recurrent localized prostate cancer. These attributes make HIFU especially promising for the veteran population, which tends to have higher rates of comorbidities and more complex medical needs. These complexities are due to various service-related exposures, such as burn pits, Camp Lejeune water contamination, and herbicides like Agent Orange and Agent Blue, which are associated with a higher prevalence of chronic health conditions and oncological diseases [22]. Veterans also have higher Charleson Comorbidity Index (CCI) and American Society of Anesthesiologists (ASA) scores compared to the general population, increasing the risks associated with surgical procedures and the use of general anesthesia [22].

Despite these potential benefits, there is a lack of data regarding HIFU treatment outcomes for localized prostate cancer in the veteran population. Hence, we aim to report oncologic, complication, and functional outcomes after primary and salvage HIFU for localized prostate cancer in the veteran population. To our knowledge, this paper presents the initial and largest United States retrospective case series of HIFU use for primary and salvage treatments of localized prostate cancer in veterans.

## 2. Materials and Methods

This study was a retrospective analysis approved by the Institutional Review Board (IRB) to evaluate outcomes for patients who underwent HIFU at the Michael E. DeBakey Veterans Affairs Medical Center between 2018 and 2022. Data were acquired from electronic medical records, including demographic and clinical information, with patient comorbidities assessed using the Charlson Comorbidity Index [23].

Our study retrospectively grouped patients into two cohorts, i.e., those receiving HIFU as primary therapy and those receiving HIFU as salvage therapy following prior treatment failure. High- and low-risk patients were classified based on PSA, Gleason Score, and clinical stage per NCCN and D’Amico criteria [24,25]. HIFU was delivered to the patient utilizing the Sonablate^®^ device. (Sonablate Corp. Charlotte, NC, USA)

Patients initially underwent multiparametric MRI (mpMRI), and DynaCAD was used to analyze the images and evaluate the tumor. The UroNAV system (Philips, Amsterdam, The Netherlands) was then used to precisely target the malignant lesions. HIFU was then performed using the Sonablate Transrectal Probe in conjunction with the Sonasource Platform System. Follow-up studies included serial PSA measurements at 3, 6, 12, 18, and 24 months, as well as mpMRI or repeat prostate biopsy at 12 months, or at any time for cause, due to rising PSA.

The primary endpoints of this study were oncological outcomes that were measured using prostate-specific antigen (PSA) metrics: baseline PSA, PSA nadir, and percentage PSA decrease after ablation. Treatment failure was defined as persistent in-field tumor visualized on prostate MRI. Local recurrence was determined by biopsy-proven prostate cancer in the prostatic bed. Post-HIFU treatments, including salvage therapy, were also documented.

The secondary endpoints were functional outcomes assessed through questionnaires. Sexual function was evaluated using the Sexual Health Inventory for Men (SHIM), and urinary function was measured using the American Urological Association Symptom Score (AUASS), uroflowmetry, and post-void residual measurement.

All complications occurring 30 days following HIFU treatment were recorded and categorized according to the Clavien–Dindo classification system [26].

Statistical analyses were performed using JMP Pro 15, a statistical analysis software package [27] (JMP Statistical Discovery LLC, Cary, NC, USA). The percent reduction in PSA from baseline was calculated using the following formula: [(initial PSA − PSA at post-ablation month)/initial PSA]. These reductions were compared across follow-up time points using one-way analysis of variance (ANOVA) for both primary and salvage therapy groups. Changes in functional outcomes were assessed by comparing pre- and post-HIFU AUASS and SHIM scores using paired *t*-tests. A *p*-value of <0.05 was considered statistically significant.

## 3. Results

**Figure 1:**
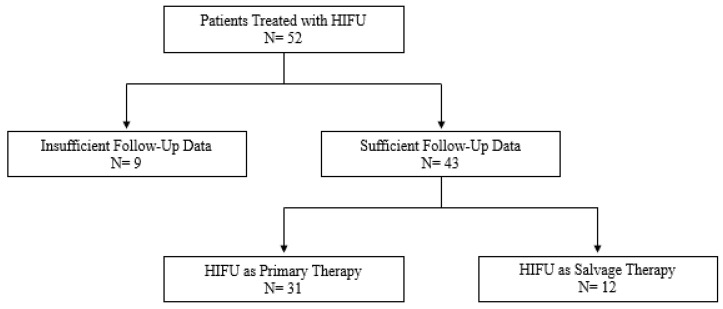
Flow chart of patients’ selection.

During the study time frame of 2018–2022, 52 veterans were treated with HIFU, and 43 had sufficient follow-up data to be included in our study. Of these, 31 patients (72.1%) received primary treatment for localized prostate cancer, and 12 patients (27.9%) received salvage therapy for localized recurrence after radiation therapy (Figure 1).

The baseline characteristics for this cohort are outlined in Table 1. The median (IQR) Charlson Comorbidity Index (CCI) was 7 for the primary treatment group and 5 for the salvage group. High-risk PCa was observed in 11 patients (35.5%) from the primary treatment group and 7 patients (58.3%) from the salvage group. All patients in the salvage group had previously undergone radiation therapy, and one had also received hormonal therapy as part of their primary treatment.

The median follow-up period was 23 months for the primary group and 25 months for the salvage group. The post-HIFU oncological outcomes are detailed in Table 2. The median PSA nadir was 0.16 ng/mL for the primary group and 0.12 ng/mL for the salvage groups. The median time to reach PSA nadir was 6 months in the primary group and 3 months in the salvage group. The median percent decrease in PSA over the follow-up period was 96% for the primary group and 98% for the salvage group. Local recurrence, identified by prostate MRI or post-ablation biopsy, was observed in five patients (16.1%) from the primary therapy group and two patients (16.6%) from the salvage therapy group.

**Figure 2:**
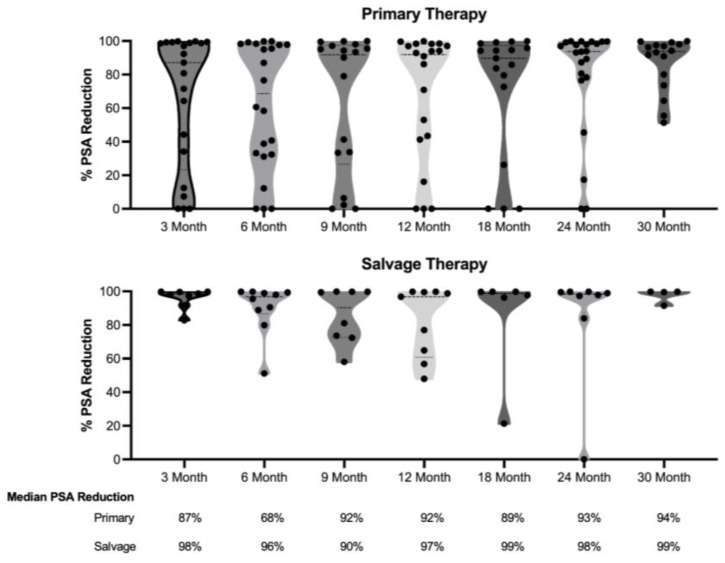
Median PSA reduction after HIFU ablation for primary (**top**) and salvage (**bottom**) therapies. Median PSA reduction = (initial PSA − PSA at post-ablation month)/initial PSA. The percent of PSA reduction from baseline was non-significant over post-ablation follow-up time for primary and salvage therapies (ANOVA, *p* = 0.3553 and *p* = 0.8057, respectively).

Figure 2 illustrates the reduction in PSA levels over a 30-month follow-up period for both the primary and salvage therapy groups. After the initial decrease measured 3 months post-therapy, there was no statistically significant change in PSA levels during the follow-up period for either group (*p* = 0.3553 for the primary group and *p* = 0.8057 for the salvage therapy group).

**Figure 3:**
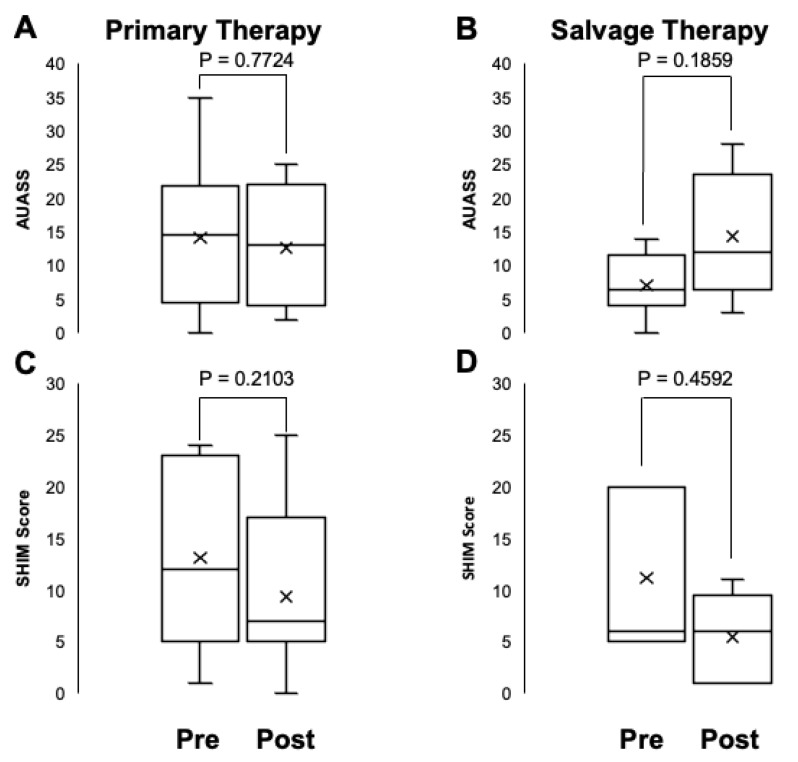
Patient-reported outcomes before and after HIFU ablation of prostate cancer, demonstrating urinary function with AUASS and sexual function with SHIM. (**A**) AUSS scores before and after primary HIFU therapy. (**B**) AUSS scores before and after salvage HIFU therapy. (**C**) SHIM scores before and after primary HIFU therapy. (**D**) SHIM scores before and after HIFU therapy.

Patient-reported urinary and sexual function were evaluated using the American Urological Association Symptom Score (AUASS) and the Sexual Health Inventory for Men (SHIM). Figure 3 displays the AUASS and SHIM scores before and after HIFU ablation for PCa. There were no statistically significant differences in AUASS or SHIM scores before and after HIFU therapy for either group (*p* = 0.2103 and 0.7724, respectively, for the primary therapy group, and *p* = 0.4592 and 0.1859, respectively, for the salvage therapy group).

Regarding complications, two patients (6.5%) from the primary treatment group experienced 30-day complications, including one case of epididymo-orchitis and one case of urethral stricture, which was managed with clinic urethral dilation. Three patients (25%) from the salvage treatment group experienced 30-day complications, including one bladder neck contracture, which required surgical incision of the bladder neck, one urethral stricture, and one case of epididymo-orchitis.

## 4. Discussion

Our retrospective review presents encouraging early and intermediate outcomes for both primary and salvage HIFU treatments of localized prostate cancer in a veteran population. The results demonstrate that HIFU can achieve significant PSA reduction, with median decreases of 96% and 98% in the primary and salvage therapy groups, respectively. These reductions remained stable over the 30-month follow-up period. Notably, HIFU appeared to preserve urinary and sexual function, as indicated by the lack of statistically significant changes in AUASS or SHIM scores post-treatment.

These findings align favorably with the 3-year outcomes of a recent study by Kaufmann et al., which reported excellent functional outcomes using focal HIFU ablation to treat PCa [17]. However, in their study of 91 patients, 21% experienced the worsening of erectile function, suggesting potential long-term declines in men’s sexual function and quality of life. Alternatively, this disparity may reflect the advancements in HIFU technology or technique. Supporting this interpretation, a phase 2 clinical trial of MRI-guided HIFU therapy for intermediate-risk PCa conducted by Ghai et al. demonstrated no significant changes in erectile function. Additionally, among the 44 patients with clinically significant PCa (GG ≥ 2) in their study, 91% of patients had no clinically significant PCa and 84% had no cancer at all 2 years post-treatment [13]. While local recurrence was observed in approximately 16% of patients in both groups, the overall complication rates were relatively low, particularly for the primary HIFU group. This aligns with findings from a prospective multicenter study of 98 men, in which 35.7% of patients reported adverse events, such as urinary tract infection or urinary retention, following HIFU treatment [28,29]. Overall, our findings suggest that HIFU may offer a viable treatment option for veterans with localized prostate cancer, including those with high-risk disease or those requiring salvage therapy.

A key finding of our study is the comparable efficacy of HIFU in both primary and salvage settings. Despite the inherent challenges of treating patients who have undergone previous radiation therapy, the salvage group achieved similar PSA reduction percentages and local recurrence rates to the primary HIFU group. This is particularly noteworthy given that the salvage group had a higher proportion of high-risk prostate cancer cases (58.3% vs. 35.5% in the primary group). These results align with findings from a larger multicenter study by Crouzet et al., which reported promising outcomes for salvage HIFU after failed external beam radiotherapy (EBRT) [30]. Their study of 418 patients showed 7-year cancer-specific and metastasis-free survival rates of over 80%. Notably, while Crouzet et al. reported significant morbidity, particularly in earlier cases, our study found a lower complication rate of 25% in the salvage group. This suggests that advancements in HIFU technology and technique may be improving the safety profile of salvage HIFU. These results collectively suggest that HIFU may be an effective option for patients who have failed primary radiation therapy, offering a less invasive alternative to salvage radical prostatectomy, with the potential for lower morbidity than previously reported.

The PSA kinetics observed in our study provide valuable insights into the oncological response to HIFU treatment. The median time to PSA nadir was shorter in the salvage group (3 months) compared to that in the primary group (6 months), which may reflect differences in prostate tissue composition and vascularity following prior radiation therapy. These findings are broadly consistent with a systematic review by Huang et al., which analyzed 27 studies encompassing 7393 patients [31]. Their review reported mean times to PSA nadir of 2.4 to 5.4 months for whole-gland HIFU and 5.7 to 7.3 months for partial-gland HIFU. Our observed nadir times fall within these ranges, suggesting our results are in line with the broader literature. However, our study found lower PSA nadirs (a median value of 0.16 ng/mL for primary and 0.12 ng/mL for salvage therapy) compared to the systematic review’s reported means of 0.4 to 1.95 ng/mL for whole-gland and 1.9 to 2.7 ng/mL for partial-gland HIFU. This difference might be attributed to variations in patient populations, HIFU techniques, or the inclusion of salvage treatments in our study. The stability of PSA reduction over our 30-month follow-up period is encouraging, suggesting durable oncological control in the short to medium term. This contrasts with the wide range of positive biopsy rates (4.5% to 91.1% for whole-gland and 14% to 37.5% for partial-gland HIFU) reported in the systematic review, highlighting the need for standardized follow-up protocols and longer-term data. While our results are promising, longer-term follow-up will be essential to confirm the sustainability of these results and to better understand how our outcomes compare to those reported in larger, multi-institutional studies.

The differential in complication rates between primary and salvage HIFU (6.5% vs. 25%, respectively) warrants discussion. The higher rate of complications in the salvage group is expected due to the challenges of treating previously irradiated tissue [32]. Nonetheless, it is important to note that, in our study, most of these complications were low-grade and easily manageable with conservative measures or minor interventions. The lower complication rate in the primary group underscores HIFU’s potential as a first-line treatment option, particularly for patients who may not be ideal candidates for more invasive therapies.

The application of HIFU in a veteran population is of particular interest due to the unique medical challenges this group faces. Veterans often present with complex medical conditions and a higher comorbidity burden compared to the general population, as indicated by a median CCI of 7 in our primary HIFU group [22,33]. This elevated comorbidity burden, which can include prior abdominal surgeries or other contraindications, often excludes veterans from being candidates for major invasive procedures, such as radical prostatectomy. The presence of small bowel near the prospective radiation field can also preclude the use of radiotherapy as primary therapy in the VA population. Despite these challenges, HIFU was well tolerated and had acceptable complication rates, highlighting its potential as an effective treatment option for veterans. Future studies in patients with significant comorbidities or surgical contraindications may provide further insights into the safety, efficacy, and applicability of HIFU in this unique patient population.

Our study has several limitations that should be acknowledged. The follow-up period (median: 23–25 months) is relatively short, particularly for assessing long-term oncological outcomes in PCa. Additionally, the sample size, while substantial for an initial series, is still modest, especially for the salvage therapy group. Larger, multi-center studies with longer follow-up periods will be necessary to confirm and extend our findings.

Long-term follow-up data will also be crucial for establishing the durability of oncological control and functional preservation. Larger studies comparing HIFU to other treatment modalities in veteran populations would provide valuable comparative effectiveness data. Furthermore, research into predictive factors for HIFU success or failure could help refine patient selection criteria and improve outcomes.

Future studies should consider incorporating mpMRI and Apparent Diffusion Coefficient (ADC) values in diagnosing clinically significant PCa. A 2017 study found that an ADC threshold of 0.747 × 10^−3^ mm^2^/s could effectively distinguish between a Gleason Score of 6 and ≥7, with an accuracy of 84%, compared to 63.6% for PI-RADS scores of ≥3 [34]. Additionally, emerging modalities like 68Ga-PSMA PET/CT have demonstrated enhanced precision in detecting PCa recurrence following surgery or radiotherapy [35]. Thus, both ADC and PSMA PET/CT offer additional data and biomarkers that could improve the detection of clinically significant PCa and provide a more comprehensive evaluation of patient outcomes.

However, many of the treatments were performed prior to the wide availability of PSMA PET/CT scanning, explaining the reliance on PSA values for follow-up. But, as this imaging modality is becoming the new standard of care, its use in the evaluation of patients with PCa prior to intervention, and in follow-up, is now advocated. This emphasizes the importance of integrating advanced imaging techniques into follow-up protocols. Future randomized controlled studies could also compare the outcomes of focal therapy for PCA with robotic-assisted radical prostatectomy (RARP) and/or IMRT+/− brachytherapy, prospectively.

Another limitation of HIFU, compared to other focal therapy options, e.g., cryotherapy, urethral-based focused ultrasound, transperineal laser ablation, etc., is the inability to treat cancers in the anterior prostate of large glands, >4 cm in anterior–posterior height, due to the maximum focal length of the treatment transducer. Addressing this limitation through advancements in technology could broaden the applicability of HIFU.

Despite these limitations, our results have important implications for clinical practice. HIFU appears to be a viable option for both primary and salvage therapies in veterans with localized prostate cancer. The preservation of functional outcomes, coupled with acceptable oncological control, makes it an attractive alternative to more invasive treatments, particularly for patients with significant comorbidities or those seeking to avoid the side effects associated with radical therapies.

## 5. Conclusions

Our review demonstrates that HIFU is a promising treatment option for localized prostate cancer in veterans, showing effectiveness in both primary and salvage therapy settings. The combination of significant PSA reduction, preservation of urinary and sexual function, and acceptable complication rates supports the continued exploration and refinement of this technique. However, for the vast majority of patients presenting with unfavorable intermediate- and high-risk localized prostate cancer, we generally advocate either RARP or IMRT, or both, as a significant number of known modalities are not advisable or acceptable. As we accumulate more long-term data and experience, HIFU may become an increasingly important tool in our armamentarium against prostate cancer, offering veterans a less invasive option that balances oncological control with quality-of-life preservation.

## Figures and Tables

**Table 1:** Baseline characteristics.

	Primary Therapy	Salvage Therapy
No. of patients (N)	31	12
Median age (IQR)	70 (64–74)	71 (67–73)
Median cc prostate volume (IQR)	33 (24–50)	22 (16–32)
Median ng/mL PSA (IQR)	6.2 (4.3–9.0)	4.9 (4.8–8.8)
Median ng/mL/cc PSA density (IQR)	0.19 (0.09–0.29)	0.22 (0.15–0.51)
Clinical stage (%)		
T1c	23 (74.2)	10 (83.3)
T2a	1 (3.2)	0 (0.0)
T2b	2 (6.5)	1 (8.3)
T2c	5 (16.1)	1 (9.3)
ISUP grade group (%)		
1	2 (6.5)	2 (16.6)
2	11 (35.5)	2 (16.6)
3	7 (22.6)	2 (16.6)
4	6 (19.4)	0 (0.0)
5	5 (16.1)	6 (50.0)
Entry biopsy		
Median no. of cores taken (IQR)	12 (12–12)	12 (12–12)
Median pos cores (any Ca) (IQR)	4 (2–6)	4 (2–7)
Median max Ca core % (IQR)	50 (20–65)	43 (24–65)
Amount of bilateral Ca on biopsy (%)	11 (35.5)	5 (41.7)
NCCN risk group (%)		
Low	2 (6.5)	2 (16.6)
Intermediate favorable	8 (25.8)	2 (16.6)
Intermediate unfavorable	10 (32.2)	1 (8.3)
High	11 (35.5)	7 (58.3)
Prior therapy (%)		
Radiation	0 (0.0)	12 (100.0)
Hormonal therapy	0 (0.0)	1 (8.3)
Median CCI (IQR)	7 (5–8)	5 (4–6)
Median BMI (IQR)	27 (22–31)	33 (30–34)
Prior abdominal surgery (%)	9 (29.0)	2 (16.6)

**Table 2:** Oncological outcomes after HIFU.

	Primary Therapy	Salvage Therapy
Median follow-up period, in months (IQR)	23 (15–34)	25 (9–39)
Median PSA nadir (IQR)	0.16 (0.06–2.96)	0.12 (0.01–0.71)
Median time to PSA nadir, in months (IQR)	6 (3–9)	3 (3–9)
Median % PSA decrease (IQR)	96 (55–99)	98 (93–99)
Local recurrence (%)	5 (16.1)	2 (16.6)
Post-HIFU treatment (%)		
Radiation	2 (6.5)	0 (0)
Hormonal	1 (3.2)	2 (16.6)
HIFU	1 (3.2)	0 (0)
Radical prostatectomy	1 (3.2)	0 (0)

## Data Availability

A raw complete dataset is not readily available due to concern for confidentiality; however, a limited de-attributed dataset could be made available upon request.
